# *The Plant Cell* welcomes 2025 Assistant Features Editors

**DOI:** 10.1093/plcell/koaf001

**Published:** 2025-01-06

**Authors:** Nancy A Eckardt, Blake C Meyers, Pablo A Manavella

**Affiliations:** American Society of Plant Biologists, USA; The Genome Center, University of California, Davis, Davis, CA 95616, USA; Department of Plant Sciences, University of California, Davis, Davis, CA 95616, USA; Institute of Subtropical and Mediterranean Horticulture, University of Málaga, Spanish National Research Council (CSIC), 29071 Málaga, Spain

**Figure 1.**
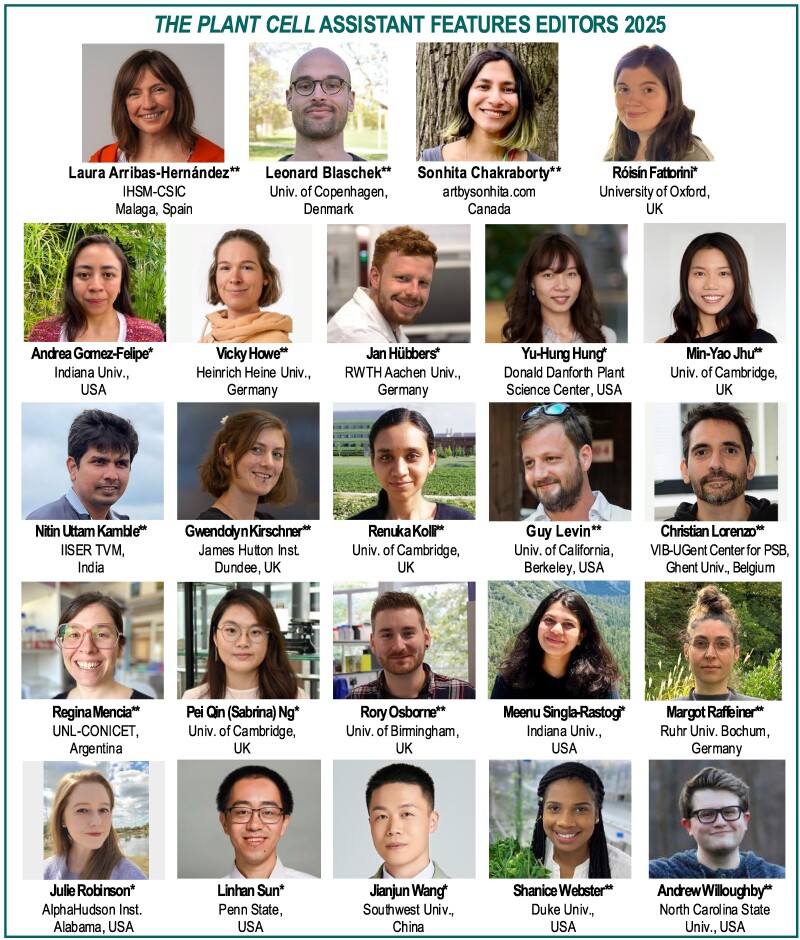
*The Plant Cell* AFEs for 2025. *Joining January 2025. **Continuing.

*The Plant Cell* is pleased to announce our Assistant Features Editors (AFEs) for 2025 (Fig. 1). *The Plant Cell* AFE program provides AFEs with experience in science writing for a broad audience, training in the peer-review process, and networking opportunities with our editorial board, authors, and other AFEs. The AFEs, in turn, deliver valuable service to the journal, our authors, and the scientific community by contributing “In Brief” article highlights and participating in editorial board activities. The AFEs gain experience and mentoring to improve their writing, receive editor and peer-review training, participate in editorial board meetings, and engage in other journal-related activities. In June of 2025, the AFEs will be supported to attend Plant Biology 2025 in Milwaukee, Wisconsin. Congratulations to our successful applicants! We are excited to work with this terrific group of early career researchers in 2025 (Fig. 1).

We strongly emphasize the writing samples and other application materials to provide evidence that an applicant shows a commitment to science communication, an aptitude for storytelling and writing in an engaging manner, and the ability to easily grasp the major findings of work published in *The Plant Cell*. We also seek to bring on a diverse group who collectively can cover the range of topics published in the journal. We accept applications for the AFE program every two years. Our next application deadline will be in September 2025 for positions to start in 2026 and 2027. Watch for the call for applications on the journal website and social media channels. *The Plant Cell* AFE program is highly competitive and a great addition to your resume.

Our sincere thanks to the AFEs stepping down in 2025 for their two years of service to the journal:

Michael Busche. University of Wisconsin, USA.

Nicolas Doll. École Normale Supérieure (ENS) de Lyon, France.

Nora Flynn. University of California, Riverside, USA.

Bradley Laflamme. University of Toronto, Canada.

Peng Liu. Donald Danforth Plant Science Center, USA.

Raul Sanchez-Muñoz, Ghent University, Belgium.

Mariana Schuster. Leibniz Institute of Plant Biochemistry, Germany.

Arpita Yadav. Penn State University, USA.

Leiyun Yang. Nanjing Agricultural University, China.

It has been a pleasure working with all of you and getting to meet in person those who could make the trip to the editorial board meeting and Plant Biology 24 in Honolulu, Hawaii in June 2024. We wish you all the best in your future endeavors.

